# Geniposide Protects against Obesity-Related Cardiac Injury through AMPK*α*- and Sirt1-Dependent Mechanisms

**DOI:** 10.1155/2018/6053727

**Published:** 2018-11-04

**Authors:** Zhen-Guo Ma, Chun-Yan Kong, Peng Song, Xin Zhang, Yu-Pei Yuan, Qi-Zhu Tang

**Affiliations:** ^1^Department of Cardiology, Renmin Hospital of Wuhan University, Wuhan 430060, China; ^2^Cardiovascular Research Institute of Wuhan University, Wuhan 430060, China; ^3^Hubei Key Laboratory of Cardiology, Wuhan 430060, China

## Abstract

Our previous study found that geniposide, an agonist of glucagon-like peptide-1 receptor (GLP-1R), protected against cardiac hypertrophy via the activation of AMP-activated protein kinase *α* (AMPK*α*). However, the effects of geniposide on obesity-related cardiac injury remain unknown. Here, we examine whether geniposide attenuates obesity-related cardiac dysfunction. Adult mice were fed a high-fat diet (HFD) for 24 weeks to induce obesity, with the last 3 weeks including a 21-day treatment with geniposide. Morphological changes, cardiac function, and remodeling were assessed. HFD-induced metabolic syndrome, featured as obesity, hyperglycemia, and cardiac hypertrophy, was prevented by geniposide treatment. Geniposide preserved cardiac function in the obese mice. Furthermore, geniposide attenuated myocardial inflammation and myocyte apoptosis induced by HFD. Geniposide activated AMPK*α* and sirtuin (Sirt1) in vivo and in vitro. *Ampkα* deficiency reversed the inhibitory effects of geniposide on cell loss. *Sirt1* deficiency abolished the inhibitory effects of geniposide on inflammation in the cardiomyocytes. Geniposide completely lost its protective effects on *Ampkα* knockout mice after *Sirt1* deficiency achieved by a nanoparticle transfection reagent. The activation of Sirt1 by geniposide was abolished by *Glp-1r* deficiency in vitro. Geniposide reverses molecular pathology and cardiac dysfunction via both AMPK*α*- and Sirt1-dependent mechanisms. Geniposide is a potential therapeutic drug for cardiovascular complications induced by obesity.

## 1. Introduction

Obesity, a major burden worldwide, induces structural and functional changes in the heart. Obesity predisposes an individual to devastating cardiac complications, such as cardiac dysfunction and heart failure [1, 2]. The pathogenesis of obesity-related cardiac injury is complex and multifactorial. The driving forces in this pathogenesis include an excess supply of saturated fatty acids, myocardial inflammation, epigenetics, hypertrophy, and loss of cardiomyocytes [3–5]. Excessive adipose tissues result in the production of inflammatory factors, causing impaired cardiac function and cardiomyocyte apoptosis [6]. Additionally, aberrant fatty acids can activate cardiac nuclear factor-kappa B (NF-*κ*B) and thus promote the production of proinflammatory cytokines [7]. Therefore, determining the inhibitory mechanisms of chronic, low-grade inflammation and cell apoptosis in the hearts of obese individuals is important.

Previous studies demonstrate a central role of AMP-activated protein kinase *α* (AMPK*α*) in obesity-induced cardiac injury. AMPK*α* can suppress the activation of NF-*κ*B, a key regulator of chronic inflammation [8]. The findings from our lab show that the activation of AMPK signaling attenuates cardiac inflammation and cardiomyocyte apoptosis in mice with diabetes [9]. Moreover, AMPK*α* deficiency exaggerates cardiac hypertrophy and contractile dysfunction caused by obesity [10], and the activation of AMPK*α* protects against cardiac remodeling due to obesity [11]. Therefore, finding AMPK activators would be of great significance to treat obesity-related cardiac dysfunction.

Geniposide (GE) is a natural product isolated from the gardenia plant. Geniposide has anti-inflammatory and antihyperlipidemia properties [12, 13]. Geniposide exerts its biological effects as an agonist of glucagon-like peptide-1 receptor (GLP-1R) [14, 15]. Moreover, our previous study found that geniposide attenuated pressure overload-induced cardiac remodeling via the GLP-1R/AMPK*α* pathway [15]. However, the potential effects of geniposide on inflammation and apoptosis in overnutrition-induced cardiomyopathy are still unknown. Here, we have shown that geniposide improves cardiac function in obese mice via both AMPK-dependent antiapoptotic action and sirtuin- (Sirt1-) dependent anti-inflammatory action.

## 2. Method and Materials

### 2.1. Reagents

Geniposide was purchased from Shanghai Winherb Medical Science Co. (Shanghai, China). The purity of geniposide was above 98% as determined by HPLC analysis. Antibodies against p-NF-*κ*B (1 : 1000 dilution), NF-*κ*B (1 : 1000 dilution), *β*-actin (1 : 1000 dilution), Bax (1 : 1000 dilution), Bcl-2 (1 : 1000 dilution), cleaved caspase 3 (1 : 1000 dilution), acetyl-CoA carboxylase (ACC, 1 : 1000 dilution), p-ACC (1 : 1000 dilution), extracellular regulated protein kinases (ERK, 1 : 1000 dilution), and p-ERK (1 : 1000 dilution) were purchased from Cell Signaling Technology (Danvers, Massachusetts, USA). Proliferating cell nuclear antigen (PCNA, 1 : 200 dilution) was obtained from Santa Cruz (Dallas, TX, USA). Sirt1 (1 : 1000 dilution) and GLP-1R (1 : 1000 dilution) were obtained from Abcam (Cambridge, UK). We used the BCA protein assay kit from Pierce (Rockford, IL, USA) to determine protein concentrations. Palmitic acid (PA) was also obtained from Sigma-Aldrich (St. Louis, MO, USA).

### 2.2. Animals and Animal Model

All the animal experiments were performed in accordance with the National Institutes of Health guidelines (NIH Publication, revised 2011) and the guidelines of the Animal Care and Use Committee of Renmin Hospital of Wuhan University. The animal studies also follow the ARRIVE guidelines. Male C57/B6J mice (age: 8–10 weeks; body weight: 25.5 ± 2 g) were purchased from the Institute of Laboratory Animal Science at the Chinese Academy of Medical Sciences (Beijing, China) and housed for more than 1 week before experimentation. After that, the mice were grouped according to a random number table and fed a high-fat diet (HFD, 45% kilocalories from fat, Institute of Laboratory Animal Science at the Chinese Academy of Medical Sciences, D12451, composition: protein 20 kcal%, carbohydrate 35 kcal%, and fat 45 kcal%) or a normal diet (ND, 10% kilocalories from fat) for 24 weeks, with only the last 3 weeks including a 21-day treatment with a previously used dose of geniposide (50 mg/kg, 0.2 ml, po) or an equal volume of saline (0.2 ml, po). The blood glucose levels were measured by mandibular puncture blood sampling. At the endpoint, all the mice were sacrificed with an overdose of sodium pentobarbital (200 mg/kg, i.p.) to harvest their heart and to calculate the following ratio: heart weight (HW)/tibia length (TL). To confirm the role of AMPK*α* in geniposide-mediated cardioprotection, *Ampkα2* global knockout mice were used and subjected to HFD or ND for 24 weeks with treatment with geniposide for 3 weeks. The source of *Ampkα2* global knockout mice has been described previously [16, 17]. To verify the hypothesis that Sirt1 is involved in geniposide-mediated cardioprotection, si*Sirt1* and the si*RNA* control were delivered to the heart using a nanoparticle transfection reagent (Altogen Biosystems, NV, USA) via 3 injections (once every week) into the tail vein beginning from the initial geniposide treatment (21 weeks after HFD) [18].

### 2.3. Echocardiography and Hemodynamics

Randomisation was not carried out due to the difference of body weight after HFD. After being anesthetized with 1.5% isoflurane, the mice were subjected to detection of cardiac geometry and function using a MyLab 30CV ultrasound (Esaote SpA, Genoa, Italy) equipped with a 10 MHz linear array ultrasound transducer [9, 15–17, 19, 20]. M-mode tracings were recorded from the short axis of the left ventricle (LV) at the level of the papillary muscles. Chamber dimensions and cardiac function were measured based on at least three beats.

LV performance was measured in mice anesthetized with 1.5% isoflurane using a 1.4-French Millar catheter transducer (SPR-839; Millar Instruments, Houston, USA) that was connected to a Millar Pressure-Volume System (MPVS-400; Millar Instruments). We analyzed the obtained data using PVAN data analysis software.

### 2.4. Determination of Fasting Insulin and Lipid Metabolites

Three weeks after geniposide treatment, blood was collected from the retroorbital plexus of the mice after 6 h of fasting. Fasting insulin levels were examined by an ELISA kit (Millipore, Billerica, MA, USA). Serum triacylglycerol (TG), glycerol, and nonesterified fatty acid (NEFA) contents were examined using a TG assay kit (E4506, BioVision, California, USA), a free glycerol colorimetric assay kit (K634, BioVision), and a NEFA assay kit (K612-100, BioVision), respectively.

### 2.5. Morphometric Analyses, ELISA Detection, and TUNEL Staining

Hearts harvested from the sacrificed mice were arrested in 10% KCl, fixed in 4% paraformaldehyde overnight, and subsequently processed for paraffin embedding and sectioning into 5 *μ*m slices. After hydration via a graded ethanol series, the sections were stained with hematoxylin and eosin (H&E) to outline the gross heart or picrosirius red (PSR) to determine the average collagen volume. Digital images of the slides were then captured by Photo Imaging System (H550L; Nikon, Tokyo, Japan). Each slide was blindly examined by two authors. The cross-sectional area and average collagen volume were determined by Image-Pro Plus 6.0 (Maryland, USA). In each group, 5 mice with 25 fields were used to count the cross-sectional area of cardiomyocytes. To evaluate the collagen volume, more than 30 fields in 5 mice per group were assessed.

Myocardial TNF-*α* levels were determined using an ELISA kit (#BMS607HS, Invitrogen, Carlsbad, CA) in accordance with the manufacturer's instructions. Sirt1 activity was determined using a commercial kit (ab156065) obtained from Abcam following the manufacturer's protocol.

We qualitatively analyzed myocardial apoptosis by terminal deoxynucleotidyl transferase-mediated dUTP nick-end labeling (TUNEL) staining according to the manufacturer's instructions [9, 17]. The images were quantified by Image-Pro Plus 6.0.

### 2.6. Cell Culture and Treatment

Neonatal rat cardiomyocytes (NRCMs) were prepared and cultured as previously described [21–23]. Cells were seeded in DMEM (Gibco, California, USA) supplemented with 10% FBS (Gibco). NRCMs exposed to either PA (500 *μ*mol/l) or vehicle (0.1% DMSO) were treated with geniposide (50 *μ*mol/l) for 24 h to detect the alteration in inflammatory factors. To detect myocyte apoptosis, NRCMs were cultured for 48 h. To confirm our hypothesis that AMPK*α* is involved in geniposide-induced protection, cardiomyocytes were infected with adenoviral vectors carrying *Ampkα*2 small hairpin RNAs (sh*Ampkα*) or the scrambled sh*RNA* at an MOI of 100 for 4 h. Sh*Ampkα* and the scrambled sh*RNA* were used in our previous studies [9, 15–17]. To inhibit the activity of Sirt1, the cells were transfected with si*Sirt1* or a scrambled RNA using Lipofectamine 2000 (Invitrogen) as described previously [17]. To verify the hypothesis that the activation of Sirt1 is induced by the treatment of geniposide, cardiomyocytes were incubated with Ex9-39 (10 *μ*M) for 24 h. To knock down *Glp-1r*, cardiomyocytes were transfected with si*Glp-1r* or a scrambled RNA using Lipofectamine 2000 for 4 h. To detect the level of NAD+, cardiomyocytes were incubated with PA for 24 h after *Glp-1r* knockdown; after that, the cells were collected for further detection using an NAD/NADH quantitation colorimetric kit (Zurich, Switzerland).

### 2.7. Western Blot Analysis and Quantitative Real-Time PCR

Frozen left ventricles were pulverized, and total and nuclear proteins were extracted using a Nuclear and Cytoplasmic Protein Extraction Kit obtained from Beyotime Institute of Biotechnology (Beijing, China). Then, the proteins were loaded into 10% SDS-PAGE gels and subsequently electrotransferred to PVDF membranes (Millipore). Samples were incubated with primary antibodies overnight at 4°C and secondary antibodies for 1 h at room temperature. These membranes were scanned by a two-color IR imaging system (Odyssey, LI-COR). The intensity of the band was normalized to that of *β*-actin.

Total RNA was isolated using TRIzol reagent (Invitrogen) and was reverse transcribed into cDNA using the Transcriptor First Strand cDNA Synthesis Kit (Roche, Basel, Switzerland). The target genes were amplified using LightCycler 480 SYBR Green 1 Master Mix (Roche, 04707516001). The mRNA levels were normalized to those of *β*-actin. The primers were reported in our previous studies [9, 15–17, 19, 20].

### 2.8. Cell Viability and Caspase 3 Activity Detection

Cell viability was determined using a commercial kit obtained from Dojindo Molecular Technologies (Rockville, MD, USA) following the manufacturer's protocol. To detect the activity of caspase 3, the protein lysates were centrifuged (4230*g*, 10 min) to collect the supernatant fractions. We measured caspase 3 activity using a kit obtained from Beyotime Biotechnology (Shanghai, China).

### 2.9. Sirt1 Activity Detection

#### 2.9.1. Statistical Analysis

The group sizes of the experiments in this study were estimated based on power analysis of HW/TL with an *α* error of 5% and a power of 80%, which is consistent with our previous article [15]. All the data are expressed as the mean ± SD and analyzed with SPSS software (version 22.0). The raw data were assessed independently by two authors to ensure the correctness of the conclusions. Data for normality were tested using the Kolmogorov-Smirnov test. Two groups were compared using two-tailed Student's *t*-test. Multiple comparisons were performed by one-way analysis of variance (ANOVA) followed by Tukey's test for post hoc analysis. *P* < 0.05 was considered significant.

## 3. Results

### 3.1. Geniposide Alleviated Obesity-Related Cardiac Injury

**Figure 1:**
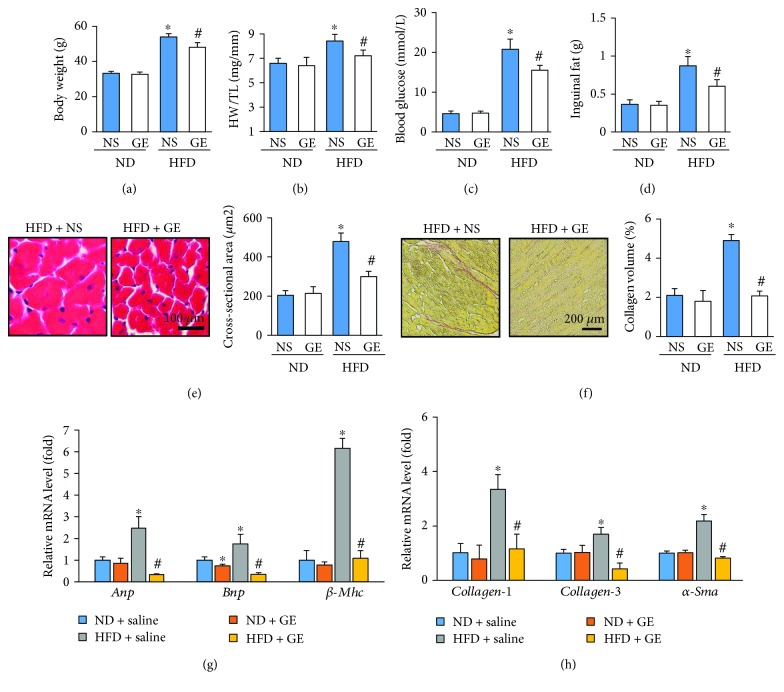
Attenuation of obesity-related cardiac injury in mice treated with geniposide (50 mg/kg) for 3 weeks. (a, b) Summary data of body weight and heart weight/tibia length (*n* = 10). (c) The blood glucose in the indicated groups (*n* = 10). (d) The inguinal fat weight (*n* = 10). (e) Representative pictures of H&E staining and quantification of the cross-sectional area (*n* = 5). (f) Representative pictures of PSR staining and quantification of collagen volume (*n* = 5). (g) The mRNA expressions of hypertrophic markers (*n* = 6). (h) The mRNA expressions of fibrotic markers (*n* = 6). The data are expressed as the mean ± SD. ^∗^ *P* < 0.05 (versus ND + NS); ^#^ *P* < 0.05 (versus HFD + NS). The data were compared by one-way ANOVA with Tukey's post hoc analysis.

Chronic HFD intake for 24 weeks overtly increased body weight (BW) and HW/TL. These changes were attenuated in mice treated with geniposide (Figures 1(a) and 1(b)). Geniposide did not affect BW or HW/TL under a normal diet (Figures 1(a) and 1(b)). Mice with HFD had increased blood glucose, inguinal fat, and fasting insulin levels, an effect that was attenuated by geniposide treatment (Figures 1(c) and 1(d), Figure S1(a)). After the 3-week geniposide treatment, reductions in serum TG, glycerol, and NEFA were observed in mice of the HFD group treated with geniposide (Figures S1(b)–S1(d)). The increased cross-sectional area and average collagen volume in the obese mice, as indicated by H&E and PSR staining, were attenuated after geniposide treatment (Figures 1(e) and 1(f)). Geniposide significantly decreased the mRNA levels of hypertrophic markers, including *Anp*, *Bnp*, and *β-Mhc*, and fibrotic markers, such as *collagen-1*, *collagen-3*, and *α-Sma* (Figures 1(g) and 1(h)).

### 3.2. Geniposide Treatment Restored Left Ventricular Function in Obese Mice

**Figure 2:**
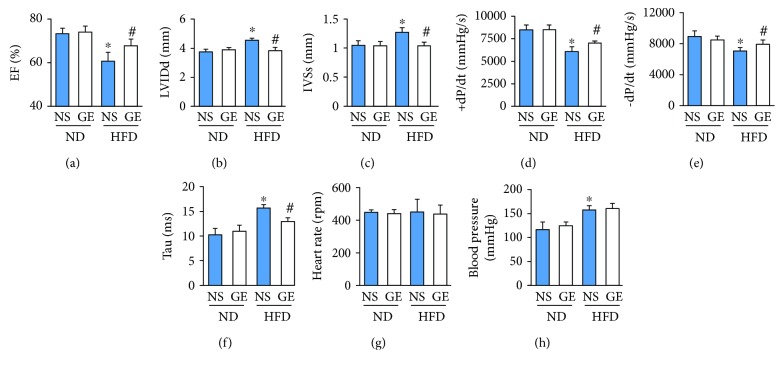
Improvement of cardiac function in obese mice after geniposide (50 mg/kg) treatment for 3 weeks. (a) Ejection fraction (EF) (*n* = 8). (b, c) Left ventricular internal diastolic diameter (LVIDd) and interventricular septum thickness at end-systoles (IVSs) (*n* = 8). (d, e) Alteration in +dP/dt and −dP/dt (*n* = 8). (f) Alteration in Tau (*n* = 8). (g) Heart rate (*n* = 8). (h) Blood pressure (*n* = 8). The data are expressed as the mean ± SD. ^∗^ *P* < 0.05 (versus ND + NS); ^#^ *P* < 0.05 (versus HFD + NS). The data were compared by one-way ANOVA with Tukey's post hoc analysis.

As shown in Figure 2, the mice fed the HFD exhibited cardiac dysfunction as indicated by the dramatic decrease in ejection fraction (EF) compared with those in mice fed the ND (Figure 2(a)). HFD resulted in an increased left ventricular internal diastolic diameter (LVIDd) and interventricular septum thickness at end-systoles (IVSs) (Figures 2(b) and 2(c)). However, the cardiac dysfunction caused by obesity was attenuated by treatment with geniposide (Figures 2(a)–2(c)). HFD also resulted in decreased indices of LV systolic contractile function (EF and +dP/dt) and impaired diastolic performance (decreased −dP/dt and increased Tau), which were prevented by the treatment with geniposide (Figures 2(d)–2(f)). There was no difference in the heart rate and blood pressure between the HFD + vehicle and HFD + geniposide groups (Figures 2(g) and 2(h)).

### 3.3. Geniposide Treatment Inhibited Myocardial Inflammation in Mice with HFD

**Figure 3:**
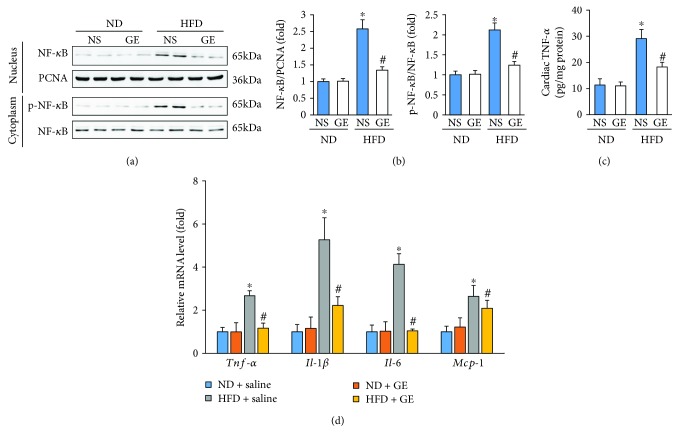
Geniposide (50 mg/kg) treatment for 3 weeks attenuated myocardial inflammation in obese mice. (a, b) Representative Western blots and quantitative results of NF-*κ*B in the nucleus and cytoplasm (*n* = 6). (c) Cardiac TNF-*α* levels as detected by ELISA (*n* = 6). (d) The mRNA levels of inflammatory factors (*n* = 6). The data are expressed as the mean ± SD. ^∗^ *P* < 0.05 (versus ND + NS); ^#^ *P* < 0.05 (versus HFD + NS). The data were compared by one-way ANOVA with Tukey's post hoc analysis.

Previous research reported that geniposide inhibited the inflammatory response in primary mouse macrophages [12]. Therefore, we next sought to determine whether geniposide inhibits the inflammatory response caused by HFD. Nuclear NF-*κ*B levels in obese mice with or without geniposide were determined by Western blot. HFD-induced nuclear translocation of NF-*κ*B was inhibited by geniposide (Figures 3(a) and 3(b)). HFD-triggered upregulation of p-NF-*κ*B in the cytoplasm was lower in mice subjected to HFD + geniposide compared with that in mice in the HFD + vehicle group (Figures 3(a) and 3(b)). Next, we detected myocardial TNF-*α* expression using an ELISA kit and found that HFD enhanced myocardial TNF-*α* expression, which was attenuated by the treatment of obese mice with geniposide (Figure 3(c)). Further detection of inflammatory factors, including *Tnf-α*, *Il-1β*, *Il-6*, and *Mcp-1*, also supports the notion that geniposide attenuates HFD-induced myocardial inflammation (Figure 3(d)).

### 3.4. Geniposide Treatment Attenuated HFD-Induced Cell Apoptosis

**Figure 4:**
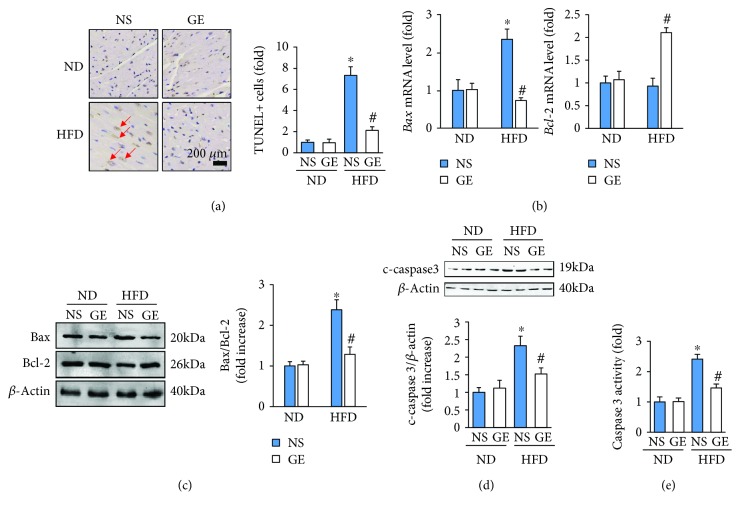
Geniposide (50 mg/kg) treatment for 3 weeks attenuated cell apoptosis in obese mice. (a) Representative images of TUNEL staining and quantitative results (*n* = 5). (b) The mRNA levels of *Bax* and *Bcl-2* (*n* = 6). (c, d) Representative Western blots and quantitative results of Bax, Bcl-2, and cleaved caspase 3 (*n* = 6). (e) The activity of cardiac caspase 3 (*n* = 6). The data are expressed as the mean ± SD. ^∗^ *P* < 0.05 (versus ND + NS); ^#^ *P* < 0.05 (versus HFD + NS). The data were compared by one-way ANOVA with Tukey's post hoc analysis.

Cell apoptosis was also involved in the development of obesity-induced cardiomyopathy [3, 24]. An increased proportion of apoptotic cells was observed in mice fed the HFD, which was reduced significantly following geniposide treatment (Figure 4(a)). The mRNA level of *Bax* was dramatically increased in the obese mice but remained lower in mice treated with HFD ± geniposide (Figure 4(b)). Geniposide upregulated the mRNA level of *Bcl-2* in mice subjected to HFD (Figure 4(b)). The inhibitory effects of geniposide on apoptosis were further confirmed by Western blot, which showed that geniposide attenuated the ratio of Bax to Bcl-2 and cleaved caspase 3 induced by HFD (Figures 4(c) and 4(d)). Though there was no difference in cardiac caspase 3 activity among mice without HFD, the relative caspase 3 activity in the heart tissue of geniposide-treated mice was lower than that in mice not receiving geniposide treatment after HFD (Figure 4(e)).

### 3.5. Geniposide Inhibited Apoptosis via AMPK*α*

**Figure 5:**
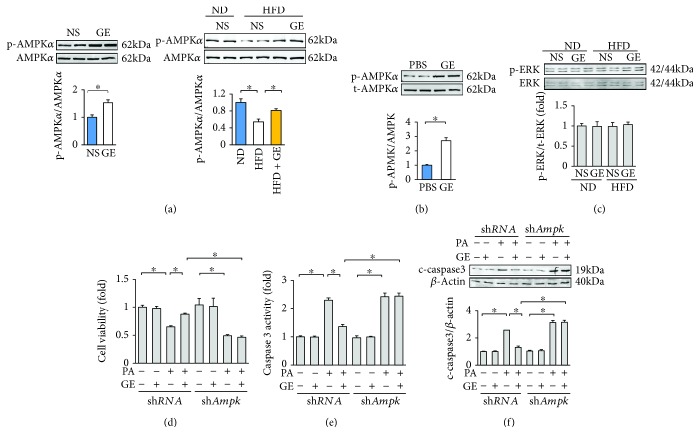
Geniposide exerted antiapoptotic action via the activation of AMPK*α*. (a) Representative Western blots and quantitative results of AMPK*α* in the hearts (*n* = 6). (b) Representative Western blots and quantitative results of AMPK*α* in the cardiomyocytes. Cardiomyocytes were treated with geniposide (50 *μ*mol/l) for 24 h to detect AMPK*α*. (c) Representative Western blots and quantitative results of ERK (*n* = 6). (d) Cell viability after geniposide (50 *μ*mol/l) for 48 h. (e) The activity of caspase 3 after geniposide (50 *μ*mol/l) for 48 h. (f) Representative Western blots and quantitative results of cleaved caspase 3 after geniposide (50 *μ*mol/l) for 48 h. All in vitro data are expressed as the mean ± SD from 5 independent experiments. ^∗^ *P* < 0.05 compared with the control. For (b), data were compared by two-tailed Student's *t*-test. For others, data were compared by one-way ANOVA with Tukey's post hoc analysis. PA: palmitic acid.

Our previous study demonstrated that geniposide could activate AMPK*α* in the heart [15]. In view of our finding that the activation of AMPK*α* protects against diabetes-related cardiomyocyte apoptosis [9], we used Western blot to assess the phosphorylation status of AMPK*α*. Consistent with our previous study, geniposide increased the phosphorylation of AMPK*α* even in mice without HFD (Figure 5(a)) [15]. Phosphorylation of AMPK*α* was decreased in the obese mice and restored after the treatment with geniposide (Figure 5(a)). Geniposide treatment also restored AMPK*α* activity, as detected by the phosphorylation of ACC in the hearts of obese mice (Figure S2(a)). In agreement with the result in vivo, geniposide activated AMPK*α* at baseline in cardiomyocytes (Figure 5(b)). We previously showed that geniposide affects the phosphorylation of ERK in hypertrophic hearts [15]. However, in the current study, we did not observe any alteration of p-ERK in any of the groups (Figure 5(c)). To investigate the protective effects of geniposide in vitro, NRCMs were treated with PA because palmitate is markedly elevated in obese mice, and exposure of cardiomyocytes to PA produces a model of fatty acid-induced injury in vitro [25, 26]. To verify the hypothesis that the protection of geniposide is dependent on the activation of AMPK*α*, we knocked down *Ampkα* in cardiomyocytes. The efficiency of sh*Ampkα* in cardiomyocytes was detected in our previous study [9, 15, 16]. *Ampkα* mRNA level in cells that infected with sh*Ampkα* decreased to 32% of that of cells infected with sh*RNA* (Figure S2(b)). Pretreatment with geniposide improved cell viability after PA challenge, and this effect was abolished after *Ampkα* deficiency (Figure 5(d)). Further detection of cleaved caspase 3 expression and caspase 3 activity showed that geniposide attenuated the expression of cleaved caspase 3 and caspase 3 activity, and deficiency of *Ampkα* completely offsets the protective action of geniposide on cardiomyocyte apoptosis (Figures 5(e) and 5(f)).

### 3.6. Geniposide Blocked the Production of Inflammatory Factors via Sirt1

**Figure 6:**
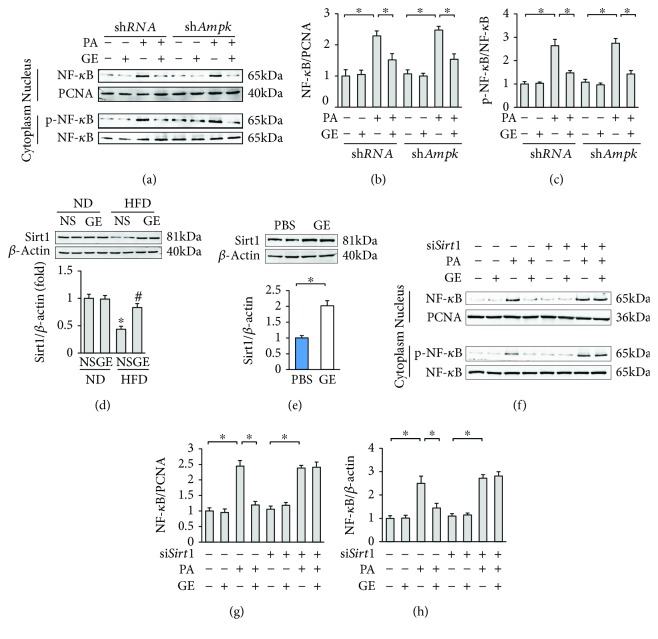
Geniposide exerted anti-inflammatory action via the activation of Sirt1. (a–c) Representative Western blots and quantitative results of NF-*κ*B in the nucleus and cytoplasm after geniposide treatment (50 *μ*mol/l) for 24 h. (d) Representative Western blots and quantitative results of Sirt1 (*n* = 6). (e) The level of Sirt1 after geniposide treatment (50 *μ*mol/l) for 24 h. (f–h) Representative Western blots and quantitative results of NF-*κ*B in the nucleus and cytoplasm after geniposide treatment (50 *μ*mol/l) for 24 h. (a–c) and (e–h) were performed in cultured cardiomyocytes. All in vitro data are expressed as the mean ± SD from 5 independent experiments. ^∗^ *P* < 0.05 compared with the control. For (e), data were compared by two-tailed Student's *t*-test. For others, data were compared by one-way ANOVA with Tukey's post hoc analysis. The concentrations of the si*Sirt1* and the control were 50 nmol/l. PA: palmitic acid.

Next, we verified whether the anti-inflammation property of geniposide was mediated by AMPK*α*. The PA-induced activation of cytoplasmic p-NF-*κ*B and NF-*κ*B nuclear translocation were blocked by the treatment with geniposide (Figures 6(a)–6(c)). However, deficiency of *Ampkα* had little or no effect on activation and nuclear translocation of NF-*κ*B in cardiomyocytes (Figures 6(a)–6(c)). Geniposide significantly decreased the PA-induced production of inflammatory factors, including *Tnf-α* and *Il-1β*, and this effect was not reversed after *Ampkα* deficiency (Figures S3(a) and S3(b)). Because NF-*κ*B was negatively regulated by Sirt1 [27], we detected the alteration of Sirt1 after geniposide treatment. HFD decreased Sirt1 expression, but this effect was markedly blocked by the treatment with geniposide (Figure 6(d)). We confirmed this finding using cardiomyocytes and found that geniposide alone increased the protein level of Sirt1 in vitro (Figure 6(e)). To evaluate Sirt1 activity, we used an ELISA kit and found that geniposide treatment also restored Sirt1 activity in the heart of obese mice (Figure S2(c)). Next, we sought to determine whether the activation of Sirt1 was responsible for the anti-inflammation property of geniposide utilizing si*Sirt1* to knock down Sirt1. The efficiency of si*Sirt1* in cardiomyocytes has previously been reported [17]. *Sirt1* mRNA level in cells infected with si*Sirt1* decreased to 48% of that of cells infected with si*RNA* (Figure S3(d)). Interestingly, geniposide lost its inhibitory effects on the PA-induced activation and nuclear translocation of NF-*κ*B after *Sirt1* knockdown (Figures 6(f)–6(h)). To validate this result further, we detected the mRNA levels of *Tnf-α* and *Il-1β*, demonstrating that such inhibitory effects of geniposide on inflammatory factors were completely lost in cardiomyocytes deficient in *Sirt1* (Figures S3(e) and S3(f)).

### 3.7. Geniposide Lost Protection in *Ampk* and *Sirt1* Double Deficiency

**Figure 7:**
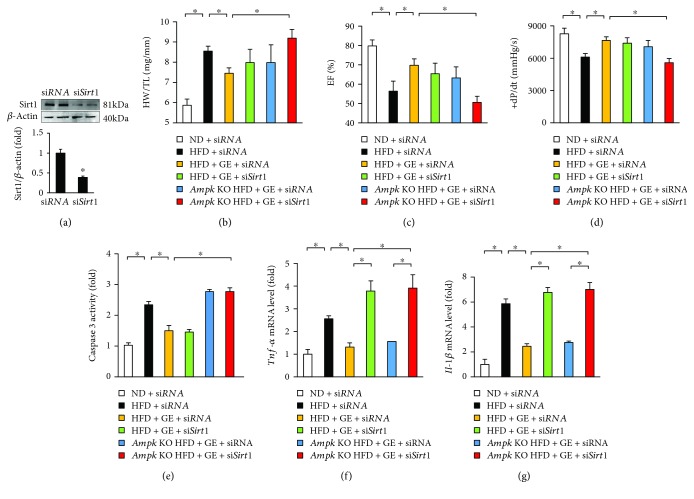
Cardiac function in *Ampk* knockout mice with geniposide. (a) The protein level of Sirt1 (*n* = 6). (b) Summary data of heart weight/tibia length (*n* = 8). (c) EF (*n* = 8). (d) Alteration in +dP/dt (*n* = 8). (e) Cardiac caspase 3 activity (*n* = 6). (f, g) The mRNA levels of inflammatory factors (*n* = 6). The data are expressed as the mean ± SD. ^∗^ *P* < 0.05 compared with the control. The data were compared by one-way ANOVA with Tukey's post hoc analysis.

To verify our hypothesis in vivo, *Ampk* global knockout mice were injected with a nanoparticle transfection reagent carrying si*Sirt1* to knock down *Sirt1* in vivo. As expected, nanoparticle-mediated transfection resulted in decreased Sirt1 protein expression (Figure 7(a)). *Ampk* and *Sirt1* double deficiency but not *Ampk* deficiency or *Sirt1* deficiency alone abolished the protection of geniposide as indicated by the alteration of HW/TL, EF, and +dP/dt (Figures 7(b)–7(d)). Geniposide lost its protection against cell apoptosis in *Ampk* knockout mice but not in the mice with *Sirt1* deficiency (Figure 7(e)). *Sirt1* deficiency but not *Ampk* deficiency abrogated the geniposide-mediated protection against myocardial inflammation (Figures 7(f) and 7(g)).

### 3.8. Geniposide Activated Sirt1 via GLP-1R

**Figure 8:**
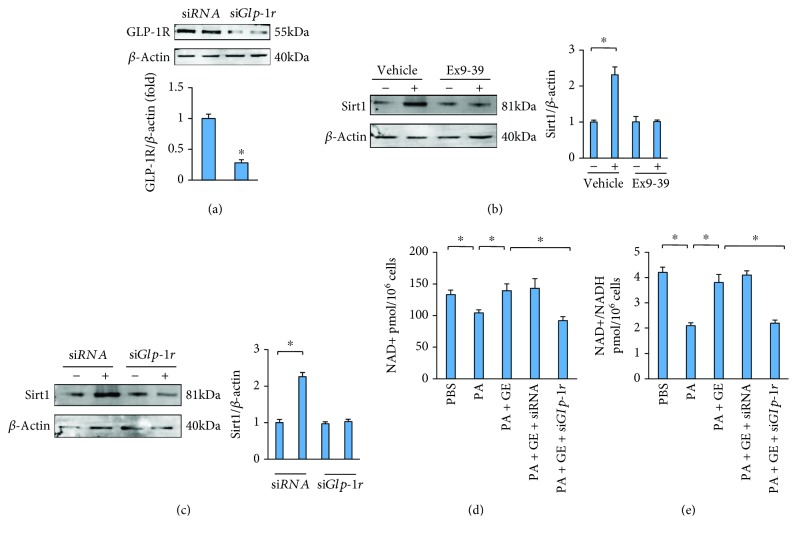
Geniposide activated Sirt1 via GLP-1R. (a) The protein level of GLP-1R. (b) Representative Western blots and quantitative results of Sirt1 after Ex9-39 treatment (10 *μ*mol) for 24 h. (c) Representative Western blots and quantitative results of Sirt1 after *Glp-1r* knockdown. (d, e) The levels of NAD+ and NAD+/NADH. All the experiments were performed in cultured cardiomyocytes, and these data are expressed as the mean ± SD from 5 independent experiments. ^∗^ *P* < 0.05 compared with the control. The data were compared by one-way ANOVA with Tukey's post hoc analysis.

We previously found that geniposide attenuated cardiac hypertrophy via GLP-1R [15]. Here, we found that geniposide does not increase the expression of Sirt1 in cardiomyocytes with GLP-1R inhibition or *Glp-1r* deficiency (Figures 8(a)–8(c)). Because Sirt1 is NAD+ dependent, we next detected whether geniposide altered the levels of NAD+ and the NAD+-to-NADH ratio in cardiomyocytes. PA resulted in decreased NAD+ and NAD+-to-NADH ratio, which were restored by treatment with geniposide (Figures 8(c) and 8(d)). The effects of geniposide on NAD+ and the NAD+-to-NADH ratio were abolished by the deficiency of *Glp-1r* (Figures 8(c) and 8(d)).

## 4. Discussion

Our previous study reported that geniposide attenuated pressure overload-induced cardiac remodeling via activating AMPK*α* [15]. However, whether geniposide inhibits obesity-related cardiac injury has remained unclear. Here, we showed that geniposide treatment protected against HFD-induced inflammatory response and cell apoptosis, thus improving cardiac function. The antiapoptotic action of geniposide was mediated by the activation of AMPK. The inhibitory action of geniposide on myocardial inflammation resulted from the activation of Sirt1.

Accumulating evidence showed that geniposide might be the “magic pill” for metabolic diseases. Lee et al. reported that geniposide enhanced lysosomal activity-regulated ER stress to attenuate hepatic dyslipidemia in rats with HFD [13]. Using the spontaneously obese diabetic mice, Kojima and his colleagues found that geniposide had an antiobesity and insulin resistance-alleviating effect [28]. Pretreatment with geniposide attenuated palmitate lipotoxicity in pancreatic *β* cells [29]. Consistent with these findings, we showed that geniposide effectively prevented body weight gain, attenuated hyperglycemia, and attenuated whole body weight in the obese mice, suggesting that geniposide exerts a robust protective effect against obesity-related metabolic dysfunction. Moreover, we showed that geniposide preserved myocardial performance and mitigated HFD-induced cardiac remodeling, which was in line with our previous report [15]. These findings support the concept that geniposide can be an effective preventive and therapeutic drug against obesity-related cardiac injury. However, one fact that cannot be ignored is that mice were treated with geniposide only for the last 3 weeks of HFD. We determined this time point because cardiac function began to decrease at 21 weeks after HFD according to our preliminary data (data not shown). Geniposide treatment beginning at 21 weeks after HFD allowed us to investigate whether geniposide attenuates advanced (preexistent) cardiac remodeling induced by obesity, which is of clinical significance. Whether geniposide can prevent cardiac alterations during the onset and development of obesity still needs further investigation.

Emerging evidence has indicated that sustained cell apoptosis caused by obesity promotes cardiac dysfunction [9, 17]. Data from our lab suggested that the inhibition of cell apoptosis resulted in the improvement of cardiac function in diabetic rats [9]. Our current study showed that geniposide attenuated obesity-induced cell loss in vivo and improved cell viability in vitro, suggesting that the reduction of apoptosis may be a potential mechanism by which geniposide exerts its protection. Our previous study reported that geniposide activated AMPK*α* and the activation of AMPK*α* reduced diabetes-related apoptosis [30]. Based on these observations, we speculated that the geniposide-elicited antiapoptotic action was mediated via the activation of AMPK*α* in obesity. Consistent with our previous finding [15], we found that geniposide activated AMPK*α* in vivo and in vitro. Moreover, transfection with sh*Ampk* abrogated the antiapoptotic action of geniposide in cardiomyocytes in response to PA treatment, implying that geniposide exerts its antiapoptotic action via the activation of AMPK.

Growing evidence implies that the production of various inflammatory factors plays pivotal roles in the process of obesity-related cardiomyopathy [31, 32]. Therefore, developing an effective strategy to suppress the overproduction of myocardial inflammation is urgently needed for obesity-induced cardiac injury. Here, we showed that geniposide attenuated the activation and nuclear translocation of NF-*κ*B in vivo and in vitro. Geniposide also prevented proinflammatory gene expression. In view of the notion that AMPK*α* blocked the activation of NF-*κ*B and the production of proinflammatory factors [9], we verified our hypothesis that the anti-inflammatory action of geniposide was dependent on the activation of AMPK*α*. Unexpectedly, *Ampkα* deficiency had no effect on the activation and nuclear translocation of NF-*κ*B or on the production of inflammatory factors in cardiomyocytes, suggesting that AMPK*α* is not involved in the protection of geniposide against myocardial inflammation. Xu et al. reported that a GLP-1R agonist promotes brown remodeling via Sirt1 [33, 34]. Moreover, Sirt1 mediates the protection of a GLP-1R agonist on HFD-induced hepatic steatosis. Geniposide is an agonist of GLP-1R [14, 15]. Therefore, we evaluated whether geniposide activates Sirt1 and found that geniposide upregulated Sirt1 expression in vivo and in vitro. Moreover, the inhibitory effects of geniposide against myocardial inflammation were completely lost in cardiomyocytes with *Sirt1* deficiency, suggesting that Sirt1 but not AMPK*α* mediated the protection of geniposide against cardiac inflammation.

Currently, there are no commercial GLP-1R agonists that can be taken orally. Here, we found that oral treatment of geniposide attenuated obesity and obesity-related cardiac injury. The previous finding from our lab showed that mice treated with this selected dose of geniposide had no hepatic injury [15]. Furthermore, geniposide has been clinically used to treat jaundice. These studies suggest that geniposide has the potential to be a clinical drug for the treatment of obesity-related cardiovascular complications.

## 5. Conclusion

Collectively, our results strongly suggest that geniposide attenuates obesity-related cardiac injury and improves cardiac function. Geniposide attenuates inflammation and cell apoptosis via activating Sirt1 or AMPK*α*. Geniposide may be of significant benefit in the treatment of obesity-related cardiac dysfunction.

## Data Availability

The data that support the findings of this study are available from the corresponding author upon reasonable request.

## Supplementary Materials

Supplementary Materials Figure S1: serum levels of insulin and lipids in mice after geniposide treatment. Figure S2: the levels of p-ACC in the hearts. Figure S3: the mRNA levels of inflammatory factors.
