# Supplementary material for: Geniposide Protects against Obesity-Related Cardiac Injury through AMPKα- and Sirt1-Dependent Mechanisms
Source: Oxid Med Cell Longev. 2018 Nov 4;2018:6053727. doi: 10.1155/2018/6053727 (PMC6247476; doi:10.1155/2018/6053727)
Supplement: Supplementary Materials — Figure S1: serum levels of insulin and lipids in mice after geniposide treatment. Figure S2: the levels of p-ACC in the hearts. Figure S3: the mRNA levels of inflammatory factors. [file 6053727.f1.docx]

**Data supplement**

**Geniposide protects against obesity-related cardiac injury through AMPKα- and Sirt1-dependent mechanisms**

Zhen-Guo Ma, Chun-Yan Kong, Peng Song, Xin Zhang, Yu-Pei Yuan, Qi-Zhu Tang

Department of Cardiology, Renmin Hospital of Wuhan University, Wuhan 430060, RP China

Cardiovascular Research Institute of Wuhan University, Wuhan 430060, RP China

Hubei Key Laboratory of Cardiology, Wuhan 430060, RP China

Corresponding author:

Qi-Zhu Tang,

Department of Cardiology,

Renmin Hospital of Wuhan University,

Cardiovascular Research Institute,

Hubei Key Laboratory of Cardiology,

Wuhan University at Jiefang Road 238, Wuhan 430060, RP China

Tel.: +86 27 88073385; Fax: +86 27 88042292. E-mail: [qztang@whu.edu.cn](mailto:qztang@whu.edu.cn)

Supplemental Methods

**
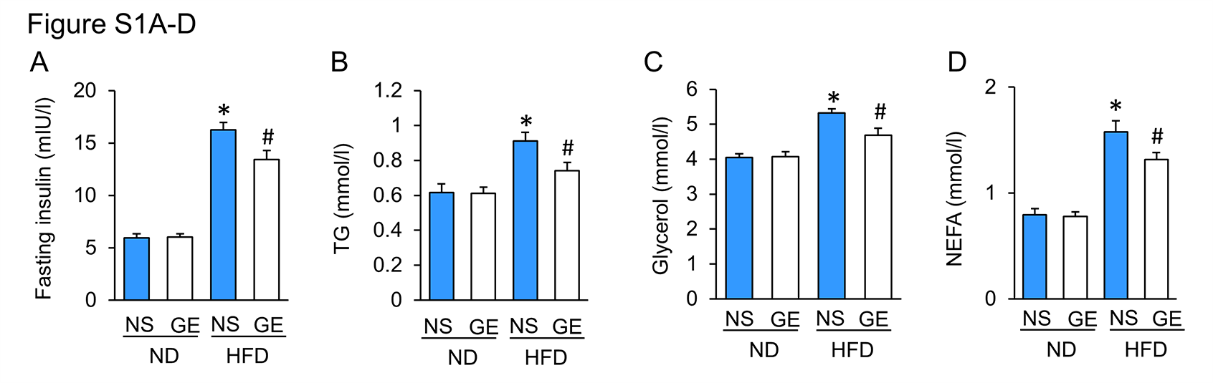
**

**Figure S1.** Serum levels of insulin and lipids in mice after geniposide treatment. A, The blood glucose in the indicated groups (n=10). B-D, Serum triacylglycerol (TG), glycerol, and nonesterified fatty acid (NEFA) contents (n=6). The data are expressed as the mean ± SD. **P* < 0.05 (versus ND+NS); #*P* < 0.05 (versus HFD+NS). The data were compared by one-way ANOVA with Tukey’s post hoc analysis.

**
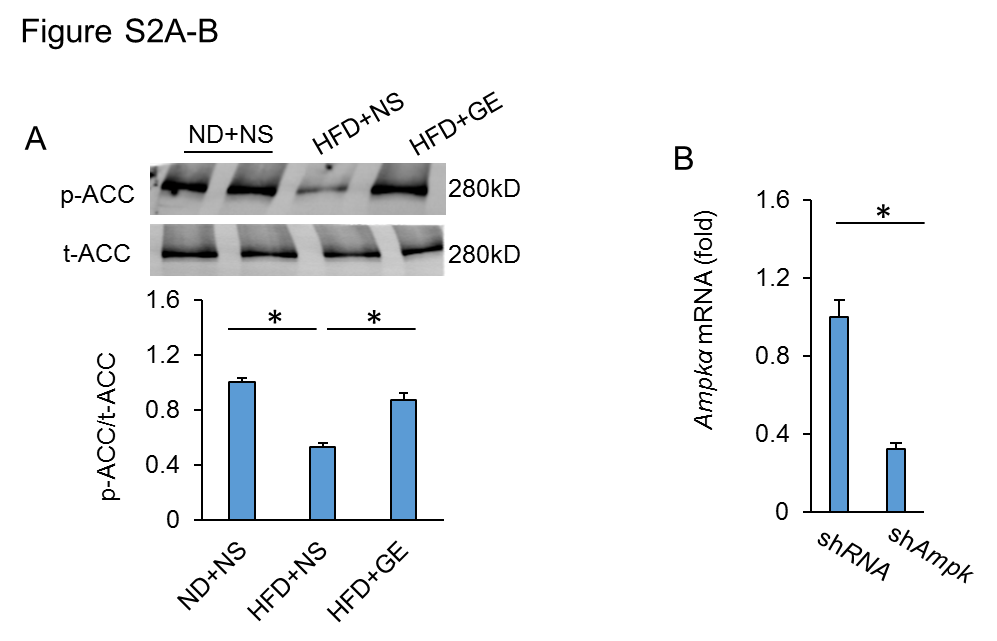
**

**Figure S2.** The levels of p-ACC in the hearts. A, Cardiac p-ACC alteration (n=6). B, The mRNA level of *Ampkα* (n=6). **P* < 0.05 compared with the control.

**
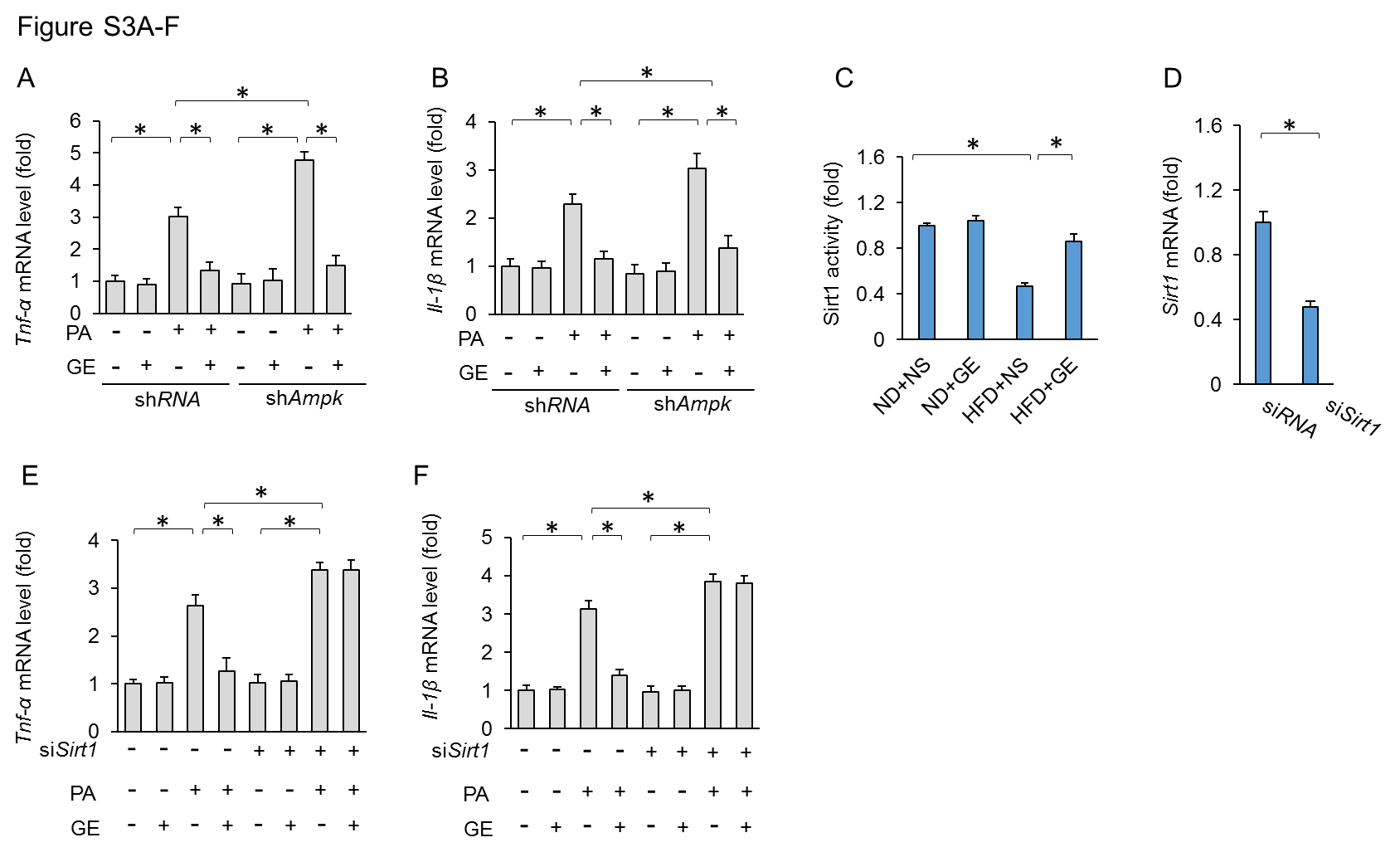
**

**Figure S3.** The mRNA levels of inflammatory factors. A-B, The mRNA level of *Tnf-α* and *Il-1β* after *Ampkα* deficiency. C, Cardiac Sirt1 activity (n=6). D, the mRNA level of *Sirt1* (n=6). E-F, The mRNA level of *Tnf-α* and *Il-1β* after *Sirt1* deficiency. For A-B and E-F, all in vitro data are expressed as the mean ±SD from 4 independent experiments. **P* < 0.05 compared with the control. Data were compared by one-way ANOVA with Tukey’s post hoc analysis.
